# Bacteriophages as Phagobiotics: Scientific Rationale and Translational Boundaries for Gut Microbiome Modulation

**DOI:** 10.3390/v18080907

**Published:** 2026-08-18

**Authors:** Fedor Zurabov

**Affiliations:** Research and Production Center Micromir, 5/23 b1, Nizhniy Kiselniy Ln., Moscow 107031, Russia; fmzurabov@gmail.com

**Keywords:** bacteriophages, phagobiotics, gut phageome, gut microbiota, microbiome modulation, phage therapy, food biocontrol, dietary supplements, functional foods, quality by design

## Abstract

Most translational work on bacteriophages has focused on antibacterial therapy or food biocontrol. A third use case is scientifically plausible but remains insufficiently defined: the intentional use of characterized phages to modulate gut microbial communities without infection-treatment claims. In this review, the term “phagobiotics” is used for defined, purified and process-controlled bacteriophages or phage cocktails intended for selective gut microbiota modulation. The concept is evaluated across natural human phage exposure, the gut phageome, mechanisms of phage-mediated community modulation, human intervention studies, preclinical models, manufacturing quality and regulatory boundaries. Current evidence supports biological plausibility and indicates that oral phage exposure can be well tolerated and, in some contexts, can selectively affect target bacterial groups without broad microbiota disruption. Generalized clinical efficacy and broad microbiome-support claims, however, remain insufficiently established. Microbiological-modulation claims require target-linked evidence, whereas claims to treat, prevent or cure disease or replace antibiotics fall outside the proposed non-therapeutic category. A proportionate framework is proposed in which natural exposure and food-use precedents inform, but do not determine, the safety rationale; product-specific controls focus on identity, purity, production-host control, manufacturing consistency, stability, genomic characterization and claim-linked evidence. Regulatory classification remains case-specific and depends on intended use, product format, target population and claims.

## 1. Introduction

Bacteriophages are the dominant viral entities in microbial ecosystems and are increasingly recognized as active participants in bacterial population dynamics, microbial evolution and ecosystem function [1,2]. In the human gut, phages constitute a major component of the virome and interact with bacterial communities at several ecological scales: strain-level host specificity, bacterial competition, community succession, mucosal organization and metabolic output [1,2,3,4]. This ecological role has renewed interest in phages not only as antibacterial therapeutics but also as potential tools for targeted manipulation of microbial communities [1,2,4].

Historically, translational phage research has been dominated by two major paradigms. The first is phage therapy: the use of bacteriophages to treat or control bacterial infections, especially where antimicrobial resistance limits conventional treatment options [5,6,7]. The second is food biocontrol: the use of phages to reduce bacterial pathogens in food matrices, food-processing environments or food-contact settings [8,9,10]. Both paradigms rely on phage specificity for bacterial hosts, but they differ in their target sites, intended uses, regulatory logic and evidentiary requirements. Phage therapy is disease-oriented and generally falls within medicinal product frameworks [6,7]. Food biocontrol is technology-oriented and focuses on reducing microbial contamination before or during food consumption [8,9,10].

**Figure 1:**
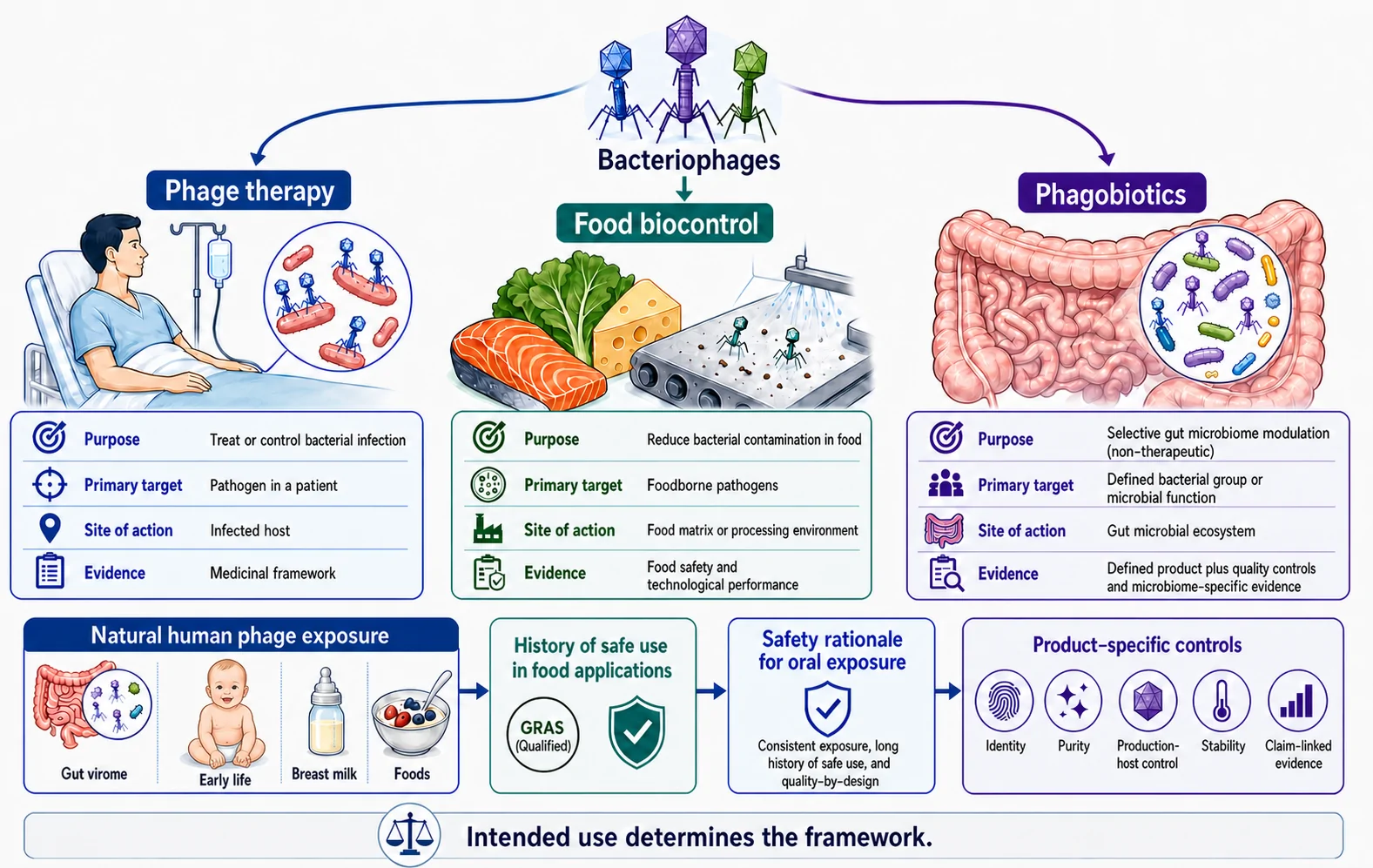
Conceptual positioning of phagobiotics among phage-based applications. Bacteriophages can be translated through distinct use frameworks depending on intended use, target site, target population and claim language. Phage therapy is disease-oriented and focuses on treatment or control of bacterial infection. Food biocontrol is technology-oriented and focuses on reducing bacterial contamination in food matrices or processing environments. Phagobiotics are proposed here as a separate gut-directed microbiome modulation concept: defined, purified and process-controlled phages or phage cocktails intended to selectively modulate gut microbiota composition or function without infection-treatment claims. Natural exposure and food-use precedents help contextualize the safety rationale for oral phage exposure, whereas product-specific controls should focus on identity, manufacturing quality, purity, stability and evidence proportionate to the intended claim. GRAS denotes “generally recognized as safe”; any GRAS conclusion is product- and use-specific and does not constitute class-wide authorization.

A third translational concept is emerging at the interface of gut virome ecology, microbiome science and translational phage biology: the use of defined bacteriophages to selectively modulate gut microbial communities without infection-treatment claims. In this review, we use the term “phagobiotics” for defined, purified and process-controlled bacteriophages or bacteriophage cocktails, preferably composed of strictly lytic and genomically characterized phages, intended to modulate the composition or functional state of the gut microbiota in a selective and controlled manner. The term is used here as a conceptual and translational framework, not as an established regulatory category. The proposed category is intentionally distinct from the established definitions of probiotics, prebiotics, synbiotics and postbiotics [11,12,13,14], and its non-therapeutic positioning remains subject to jurisdiction-specific claim rules [15,16]. This positioning is summarized in Figure 1.

Operational definition—Phagobiotics are defined, purified and process-controlled strictly lytic bacteriophages or phage cocktails intended to selectively modulate gut microbiota composition or function without claims to treat, prevent or cure disease.

Boundary conditions—The category does not include phage therapy, food-surface biocontrol, probiotics or undefined natural virome preparations. A phagobiotic product has a non-therapeutic intended use, controlled composition, characterized identity and host range, and evidence linked to a predefined microbial or host-relevant claim.

The rationale for phagobiotics rests on several converging observations. Humans are naturally and repeatedly exposed to phages through the gut ecosystem, early-life microbial colonization, breast milk, food matrices and fermented products [1,17,18,19,20,21]. Phage–bacterium interactions are therefore not artificial additions to human biology but part of the normal microbial ecology of the gastrointestinal tract. Experimental and metagenomic studies further indicate that phages can influence bacterial strain dynamics, microbial succession, mucosal ecology and community-level functions [1,17,20]. Human intervention studies remain limited, but they suggest that oral or gut-directed phage exposure can be well tolerated and may selectively affect target bacterial groups without broad disruption of overall microbiota structure under some conditions [22].

Natural and food-related phage exposure should be interpreted carefully. Background exposure does not establish efficacy, and food-use precedents should not be read as class-wide approval for gut-directed products [23,24,25]. Dose, formulation, frequency, duration, site of release and target-population context may differ substantially between incidental exposure and intentional supplementation. Natural exposure therefore provides ecological context rather than a substitute for product-specific evidence. The remaining safety questions concern whether the manufactured preparation is sufficiently defined, clean, stable, genomically characterized and supported by evidence proportionate to its intended claim [26].

Human evidence is also not yet sufficient to support broad or disease-oriented claims. Available studies provide useful signals of safety, tolerability, gastrointestinal transit, selective microbial effects and phage–microbe dynamics, but they remain modest in number, heterogeneous in design and often exploratory in endpoint structure [22,27,28,29]. Some trials show selective reduction in target bacteria without global microbiota disruption, whereas others demonstrate safe transit without clinical benefit [22,28,29]. This contrast underscores the importance of target-host presence, bacterial susceptibility and ecological context. For phagobiotics, efficacy should not be inferred from phage administration alone. It must be demonstrated through predefined microbial targets, microbiome endpoints and appropriate functional or host-relevant readouts, using product-specific studies or justified platform-bridging evidence where the product, manufacturing process, intended population and claims remain comparable.

A further challenge is that phages are not conventional dietary ingredients. Their biological activity depends on the presence, accessibility and susceptibility of bacterial hosts. They may amplify in situ, impose evolutionary pressure, select for resistant bacterial variants, alter phage–host networks and influence community-level metabolism or immune tone [30,31]. These properties make phages attractive as selective microbiome modulators, but they also mean that phagobiotics should not be treated as undefined natural extracts. The central quality questions are practical and product-specific: identity of the phage preparation, control of the production host, removal of bacterial and process-related impurities, stability under intended conditions of use, and evidence that matches the claim being made [26,32].

This review examines bacteriophages as potential phagobiotics for gut microbiome modulation from virology- and phage-product perspectives. We first summarize natural human exposure to phages through the gut virome, early-life development, breast milk and food-associated ecosystems. We then define the conceptual boundaries between phage therapy, food biocontrol and phagobiotics. Next, we review mechanisms of phage-mediated microbiota modulation, including selective predation, ecological cascades, phage-driven bacterial evolution, mucosal interactions and functional remodeling of microbial metabolism. We then evaluate human, preclinical and translational evidence relevant to gut-directed phage interventions. Finally, we propose a safety, quality and evidence framework for phagobiotic development, including a quality-by-design model that links target product profiles, critical quality attributes, manufacturing controls, microbiological and host-relevant endpoints, claim boundaries and comparability-based evidence generation.

Phagobiotics should be viewed neither as diluted phage therapy nor as food biocontrol relocated to the intestine but as a distinct gut-directed microbiome modulation concept. Their development should avoid two extremes: treating every phage-containing product as a medicinal antimicrobial and assuming that natural exposure alone is sufficient to justify microbiome-support claims. A responsible phagobiotic framework should instead combine exposure-informed safety reasoning, manufacturing quality controls and conservative, evidence-aligned claims.

## 2. Natural Human Exposure to Bacteriophages

A central premise of the phagobiotic concept is that oral exposure to bacteriophages is not biologically foreign to humans. Phages are abundant components of microbial ecosystems and are continuously encountered through the gut microbiome, early-life colonization, maternal and environmental sources, and food matrices [1,2,17,18,19]. This does not mean that any defined phage preparation can be assumed safe or effective. Rather, natural exposure provides an ecological and evolutionary context in which intentional, controlled and product-specific phage use can be evaluated. Phagobiotics should therefore be understood not as introducing a novel class of biological entities into the human gut but as attempting to standardize and direct a form of exposure that already occurs naturally.

The human gastrointestinal tract contains a diverse viral community in which bacteriophages represent a major component [1,2,3]. In this review, “phageome” denotes the bacteriophage component of the broader gut virome. Gut phages can occur as free virions, temperate prophages integrated into bacterial genomes or actively replicating lytic populations. Metagenomic, long-read and phage–host linkage methods increasingly show that phages are embedded in bacterial strain dynamics and community structure [33,34]. Their composition varies across individuals, age groups, diets, geography, disease states and detection methods [35].

Early life provides one of the strongest arguments that human exposure to phages is a normal part of microbiome development. The infant gut is rapidly colonized by bacteria, and these bacterial pioneers bring or induce phages that contribute to the developing gut virome. Longitudinal work has shown that bacteriophages can be detected very early after birth and that their dynamics are linked to bacterial colonization, maternal sources, prematurity, delivery mode and environmental exposure [17,20]. These observations matter for phagobiotics because they show that the gut microbiome does not develop in a bacteria-only ecological context. Phage–bacterium interactions are present from the earliest stages of community assembly.

Strain-resolved studies have strengthened this view. Infant gut DNA bacteriophages can persist during the first years of life rather than appearing only as transient contaminants [17]. Such persistence is relevant because it shows that phage–bacterium interactions can remain a durable feature of early microbiome assembly. The phagobiotic implication is not that infant exposure justifies phage supplementation in infants. It is that phage–bacterium dynamics are a normal and durable layer of gut ecosystem development, and any future pediatric application would require its own stringent evidence framework.

Large-scale longitudinal datasets further support the ecological relevance of phages in early life. Reconstruction of phage–bacterium dynamics from thousands of infant gut microbiome samples across multiple countries indicates that early-life phage populations are individual-specific but also follow broader patterns of ecological succession linked to putative bacterial hosts [20]. These findings move the field beyond the simple statement that “phages are present” and toward a dynamic model in which phages participate in microbial succession. For phagobiotics, this reinforces the need to evaluate phage interventions in relation to bacterial host availability and community state, not simply as fixed-dose oral exposures.

Human milk represents another route of early phage exposure. Human milk is not a sterile nutritional fluid; it contains bacterial and viral components and participates in shaping the infant gut microbiome. Reviews of human breast milk phage diversity suggest that milk may provide a physiological interface for maternal–infant phage exposure, although the evidence base remains heterogeneous and methodologically variable [18]. This route is especially relevant because it links phages not only to environmental contact but also to a nutritional and developmental context.

Dietary exposure provides a parallel route. Bacteriophages can be found in or on foods because they accompany bacterial communities in raw materials, processing environments and fermented ecosystems. Dairy products, meats, plant-derived foods and fermented products may all contain phages, although the amount and diversity of exposure depend on the food matrix, microbial load, processing, storage and detection method [19,21,36,37,38,39]. In some cases, phages are undesirable from an industrial perspective, such as dairy fermentation failures caused by phages infecting starter cultures [36,37]. In other contexts, they are intentionally used or evaluated as food safety tools against bacterial pathogens [8,9]. These examples show that phages are already part of the food–microbe interface.

Food metagenomic studies extend this picture beyond culture-based observations. Mining food metagenomes has revealed substantial underexplored diversity of double-stranded DNA bacteriophages in food-associated datasets, suggesting that conventional culture-based approaches underestimate food-associated phage diversity [19]. Broader food microbiome studies also indicate that food-associated microbial communities can overlap with human-associated microbial diversity, even though the direction and functional importance of these links require careful interpretation [21]. These findings support the ecological argument that diets may expose consumers to a broader phage repertoire than is captured by studies focused only on known foodborne pathogens or industrially problematic phages.

Fermented foods provide a particularly relevant example because they are living or microbially transformed matrices in which bacteria and phages can interact during production, maturation and storage [38]. Viral communities have been described in cheese ecosystems and other fermented products, where phages may influence bacterial succession, process stability, starter culture performance or microbial community structure [40]. Such studies are not phagobiotic studies, but they demonstrate that food-associated phage communities can be structured, persistent and functionally linked to bacterial ecology.

Food safety applications add a more controlled exposure precedent. Defined phage preparations have been evaluated or accepted for use against specific bacterial pathogens in foods, especially in the United States, and product-specific assessments have been conducted in other jurisdictions [10,23,24,25]. These uses differ from phagobiotics because their primary purpose is technological reductions in pathogen load in food matrices rather than gut microbiome modulation after ingestion. Nevertheless, they are relevant because they demonstrate that consumers may encounter intentionally applied, defined phage preparations through food under controlled conditions. This supports the general acceptability of food-related phage exposure while also underscoring the need for use-specific safety and quality assessment.

Natural exposure should not be overinterpreted. The fact that phages occur in the gut, milk and foods does not establish the efficacy of a defined phage product, nor does it determine the quality of a manufactured preparation. However, it does provide a relevant background of repeated oral exposure. Phage-specific immune responses may nevertheless be relevant depending on the product and exposure pattern [41]. For phagobiotics, the practical implication is that safety assessment can begin from a context in which humans already encounter diverse phages while focusing product-specific controls on manufacturing quality, purity, identity, stability and the intended conditions of use [26].

The relevant question is therefore not simply whether humans can encounter phages, but whether a defined manufactured preparation introduces new or avoidable risks beyond ordinary oral phage exposure. This shifts the emphasis from intrinsic concern about phages as such to the quality of the preparation, the production process, the dose, the target population and the claim. Table 1 summarizes the main natural and food-related routes of phage exposure relevant to this argument.

## 3. Phage Therapy, Food Biocontrol and Phagobiotics: Defining the Boundaries

Bacteriophages occupy different translational spaces depending on how they are used, what claims are made and what evidence is required. The same biological entity—a phage particle capable of infecting a bacterial host—may be developed as a medicinal product, a food-processing antimicrobial, a veterinary tool, an environmental biocontrol agent or a microbiome-directed product [6,7,8,9]. These categories are not interchangeable. Intended use determines the evidentiary burden, the relevant safety questions and the claim boundaries. For this reason, the phagobiotic concept should be defined not by the mere presence of bacteriophages in a product, but by the intended biological role assigned to those phages.

Phage therapy is the most familiar biomedical context. Its purpose is to treat, prevent or control bacterial infection, often in patients with established disease, recurrent infections, antimicrobial resistance or limited therapeutic alternatives. The target is usually a pathogen or opportunistic pathogen causing or contributing to disease. Product design is driven by antibacterial activity, host range, susceptibility testing, route of administration, clinical condition and therapeutic outcome. Because the intended use is disease treatment, phage therapy belongs within medicinal product logic even when regulatory pathways remain heterogeneous across jurisdictions [5,6,7,32].

Food biocontrol follows a different logic. In this setting, phages are used to reduce bacterial contamination in food matrices or processing environments, commonly targeting pathogens such as *Listeria monocytogenes*, *Salmonella*, *Shigella* or pathogenic *Escherichia coli* [10,23,24,25,42]. The intended effect occurs primarily in the food matrix before consumption, and the evidence focuses on pathogen reduction, technological performance, food safety, product identity and conditions of use. Food biocontrol precedents are important because they show that defined phage preparations can be acceptable in controlled food-related settings. However, they do not establish that phages intended to act inside the human intestine should be evaluated under the same assumptions.

Phagobiotics occupy a third conceptual space. They are not intended to treat infection, and they are not used primarily to reduce microbial contamination in a food matrix. Their intended site of action is the gut microbial ecosystem, and their intended function is selective modulation of microbiota composition or activity. This may involve reducing a defined pathobiont, shifting the abundance of a bacterial strain or group, altering a microbial functional trait, shifting a microbial configuration toward a predefined, evidence-linked state, or modulating microbial outputs relevant to gastrointestinal comfort or host physiology [1,2,4,43]. Such uses require microbiome-specific evidence rather than simple extrapolation from phage therapy or food biocontrol.

Phagobiotic activity depends on susceptible bacterial hosts, receptor availability, spatial organization, bacterial density, mucus access, coexisting microbial interactions and the evolutionary response of the target bacteria [3,33,34,35,44,45]. A fixed oral dose may undergo passive transit without replication, amplify when susceptible hosts are present, lose infectivity under gastric or bile stress, or produce effects that vary with baseline microbiota and phageome state. The relevant question is therefore not only whether phages remain infective at ingestion, but whether they reach and engage the intended target in the intended intestinal environment.

Phagobiotics also differ from probiotics, prebiotics, synbiotics and postbiotics. Probiotics are live microorganisms that confer a health benefit when administered in adequate amounts [11]. Prebiotics are substrates selectively utilized by host microorganisms that confer a health benefit [12]. Synbiotics combine live microorganisms and selectively utilized substrates [13]. Postbiotics are preparations of inanimate microorganisms or their components that confer a health benefit [14]. Bacteriophages do not fit neatly into any of these categories. They are not bacteria or yeasts; they do not act as substrates, and their activity is conditional on bacterial hosts. The term phagobiotic is therefore useful if it clarifies this distinction rather than blurring it.

The comparison with probiotics is instructive but incomplete. Both probiotics and phagobiotics may be positioned as microbiome-directed interventions, and both require evidence linking a defined product to a defined benefit in a defined population [11,12,13,14]. However, probiotics usually act through metabolic, immunological, competitive or barrier-related mechanisms of live microbial cells. Phagobiotics act through host-dependent viral infection of bacteria, bacterial lysis, ecological cascades, selection pressures and network-level changes in microbial communities [1,2,4]. A phagobiotic product should therefore be defined by product identity, label-relevant viability or potency, appropriate characterization and evidence proportionate to the intended claim rather than by analogy with probiotic enumeration alone [26].

The same caution applies to functional foods and dietary supplements. A product containing phages may be positioned in a non-medicinal category only if its intended use and claims remain non-therapeutic and if the evidence supports that positioning. Claims such as “supports microbiome balance” or “helps maintain gastrointestinal comfort” require a different evidentiary package from claims such as “treats dysbiosis”, “eradicates pathogens” or “replaces antibiotics”. The latter imply disease treatment or antimicrobial therapy and should not be used for phagobiotic products unless they are developed under appropriate medicinal frameworks [15,16].

The boundary is therefore not semantic but evidentiary. If a phage product is intended to treat infection, it should be evaluated as therapy. If it is intended to reduce pathogens in foods, it should be evaluated as food biocontrol. If it is intended to modulate the gut microbiome without disease-treatment claims, it should be evaluated through a phagobiotic framework that combines manufacturing quality controls with microbiome-specific evidence when such evidence is needed to support the intended claim.

The regulatory and evidentiary status of a phage product is determined not by the phage particle alone but by intended use, target population, claims and evidence. This principle is central to responsible phagobiotic development.

## 4. Mechanisms of Phage-Mediated Microbiota Modulation

**Figure 2:**
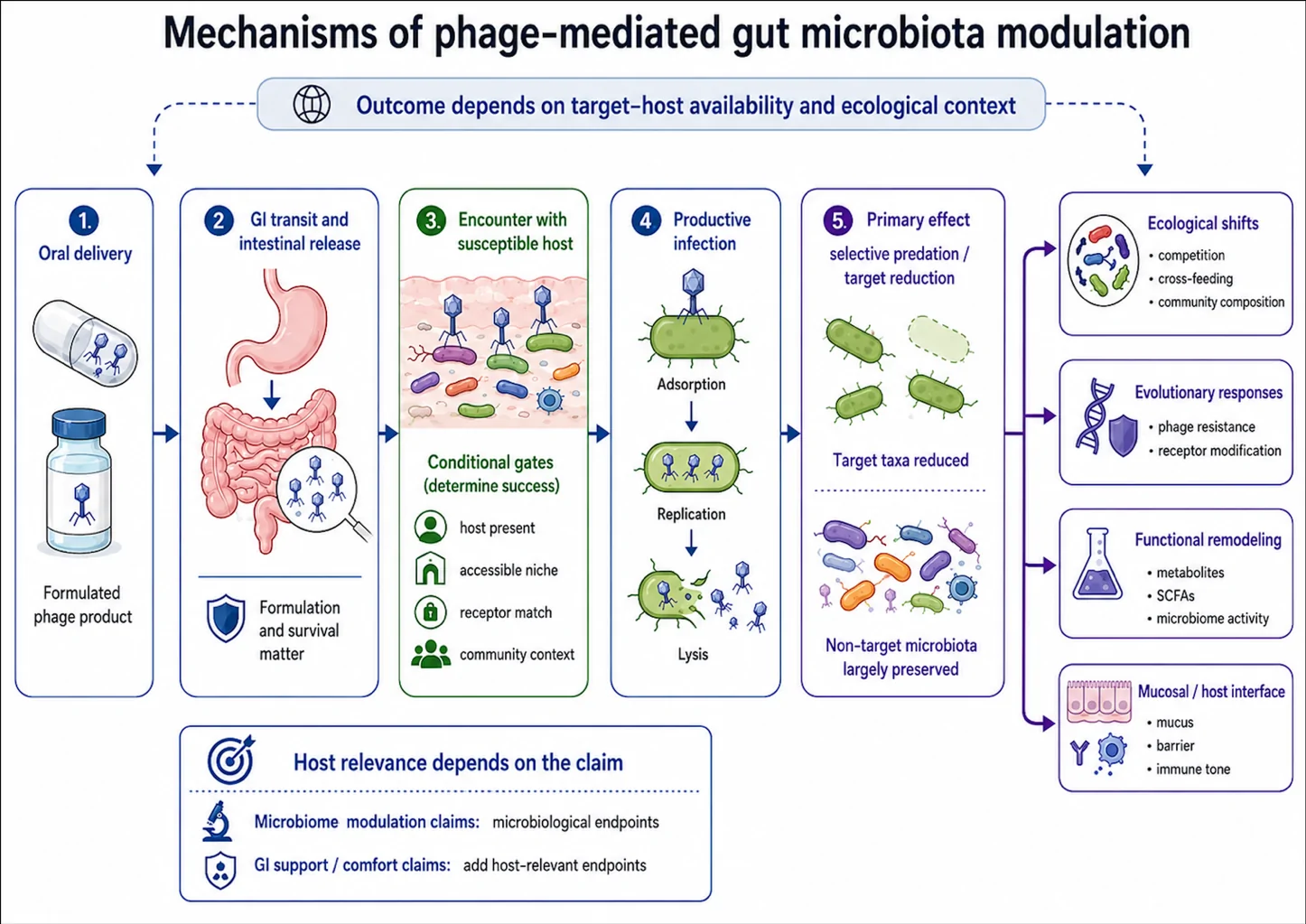
Mechanisms of phage-mediated gut microbiota modulation. Gut-directed phage activity depends on oral delivery, intestinal release, target-host availability and the ecological context of the recipient microbiota. After reaching the gut lumen, phages must encounter susceptible bacterial hosts; productive infection can drive adsorption, replication, lysis and target reduction. Downstream effects may include ecological shifts in community composition, evolutionary changes such as phage resistance or receptor modification, and functional changes in microbial metabolism or microbiome activity. These microbiological and functional effects are mechanistic evidence, but host relevance depends on the intended claim. For host-support or gastrointestinal-comfort claims, barrier or mucosal function, immune–inflammatory tone, tolerability and symptom-related endpoints may become relevant, depending on the claim and intended population. The term “survival” is used as visual shorthand for maintenance of measurable phage infectivity or biological activity during gastrointestinal transit, not organismal viability.

The rationale for phagobiotics depends on mechanisms that extend beyond simple bacterial killing. Lytic activity is central, but outcomes are shaped by target density and accessibility, mucus and luminal organization, receptor availability, resistance evolution, resident prophages and phages, immune tone, diet, formulation and baseline microbiome state [1,2,35]. A gut-directed phage may fail to encounter its host, pass through without measurable replication, reduce a target population, select receptor-modified variants or alter microbial competition and metabolism. Phagobiotics should therefore be evaluated as ecological agents acting within a recipient-specific microbiota and phageome. Figure 2 summarizes this conditional mechanism.

The most direct mechanism is selective predation. Strictly lytic phages can infect susceptible bacterial hosts, replicate intracellularly and lyse target cells, thereby reducing the abundance of specific bacterial populations [44,45]. This selectivity distinguishes phages from broad-spectrum antimicrobials and is central to the phagobiotic concept. In principle, a phage cocktail could reduce a pathobiont, antimicrobial-resistant colonizer or strain carrying an undesirable functional trait while preserving unrelated members of the microbiota. In practice, the outcome depends on host range, bacterial density, receptor expression, spatial access and the community context in which the target population exists [3,33,34,46].

Testable implication—If selective predation is the primary mechanism, a study should prospectively verify target presence and susceptibility and should measure target reduction as a principal microbiological endpoint.

Selective predation can also generate ecological cascades. Removing or suppressing one bacterial population may release resources, alter competition, modify cross-feeding networks or change the abundance of secondary taxa. These indirect effects may be beneficial, neutral or undesirable depending on the target and community state [2,3,4,43,47,48]. For example, reducing a pro-inflammatory pathobiont may permit expansion of commensal taxa and improve local metabolic output. Conversely, removal of a bacterial population could create a niche for another undesirable organism if ecological redundancy and community resilience are not considered. Phagobiotic development therefore requires more than target reduction; it requires assessment of community-level consequences.

Testable implication—If ecological cascades are expected, target reduction should be accompanied by community-wide and functional measurements capable of detecting both intended and unintended shifts.

The “kill-the-winner” model provides one useful ecological lens, but it should not be treated as a complete explanation for gut phage dynamics. In open microbial ecosystems, abundant bacterial populations may become preferential targets for phages, contributing to diversity maintenance [47,48]. The gut, however, is not a homogeneous aquatic system. It is spatially structured, host-associated, nutrient variable and immunologically active. Bacteria are embedded in mucus, biofilms, food particles and luminal gradients; phages are subject to gastric acidity, bile, digestive enzymes, mucus interactions and transit kinetics [49,50,51]. These factors make gut phage ecology more conditional than simple abundance-driven predation models imply.

Phage-driven bacterial evolution is another central mechanism. Bacteria can become resistant to phages through receptor loss or modification, restriction–modification systems, CRISPR-Cas immunity, abortive infection and other defense mechanisms [44,45]. Resistance is often treated as a limitation of phage-based interventions, but it may also be part of the desired biological effect. Receptor changes can impose fitness costs, reduce colonization capacity, alter virulence, modify capsule or lipopolysaccharide structure, or affect antibiotic susceptibility [52,53]. In a phagobiotic context, these evolutionary effects may matter as much as immediate bacterial lysis. A product designed only to maximize short-term killing may not have the same ecological outcome as one designed to channel bacterial escape toward less harmful phenotypes.

Testable implication—If resistance is predictable, studies should track receptor or susceptibility changes and determine whether escape produces loss of activity, a fitness trade-off or altered community effects.

Host range and cocktail design are mechanistically linked. Narrow-host-range phages can maximize strain-level selectivity but may leave non-susceptible target strains unaffected; broader-host-range phages or cocktails can improve coverage but may reduce ecological precision and increase manufacturing and analytical complexity [44,45,46]. A single phage may be adequate when the target strain and receptor are well defined, whereas cocktails may be preferable when target diversity or resistance escape is expected. Larger cocktails are not automatically superior. Component selection should consider complementary host range, receptor diversity, lytic performance, genome safety, inter-phage compatibility, stability and the ability to quantify component-specific potency.

Temperate phages, resident prophages and the background gut phageome complicate targeted intervention. Prophage induction can alter bacterial fitness, horizontal gene transfer, virulence and community composition, while resident phages may compete with, complement or interfere with administered phages [30,31]. This ecological importance does not justify using temperate phages in a defined product. Candidate product phages should preferentially be strictly lytic and should undergo genome-based screening for lysogeny functions, known toxin or virulence genes, antimicrobial resistance cargo and undesirable mobile elements [26,30,31,32]. Baseline and follow-up phageome characterization may be warranted when the claim or mechanism depends on interactions with resident phages or prophages.

Testable implication—If resident phages or prophages modify product performance, baseline phageome features and longitudinal phage–host linkage should predict or explain target engagement.

Spatial organization in the gut may determine whether phage–bacterium interactions occur at all. The bacteriophage adherence to mucus model proposed that phages can interact with mucosal glycoproteins and contribute to a non-host-derived layer of antibacterial defense at mucosal surfaces [49,54]. Whether and how this model applies across different gut regions, phage types and host states remains an active question. For phagobiotics, the broader implication is that phage delivery should not be understood only as maintenance of infectivity through the stomach. Relevant delivery includes localization, retention, diffusion through mucus, access to bacterial microcolonies and interaction with biofilm-like structures [50,51].

Phages may also influence the host indirectly through microbial functional remodeling. By reducing or reshaping bacterial populations, phages can alter fermentation products, short-chain fatty acids, bile acid metabolism, amino acid metabolism, gas production, redox state or inflammatory signaling [4,43,55]. These functional outcomes are especially relevant when non-therapeutic microbiome-support claims extend beyond simple microbial-modulation claims. In such cases, taxonomic endpoints are more informative if paired with functional readouts, including metagenomics, metatranscriptomics, metabolomics or targeted biomarkers, when feasible.

The host immune interface adds another layer of context dependence. Phages do not infect human cells, but phage particles and phage-mediated bacterial lysis can interact with mucus, epithelial barriers, innate recognition pathways, cytokine responses and adaptive anti-phage antibodies [41,56,57]. These interactions cannot be classified as uniformly beneficial or detrimental: their direction and magnitude depend on the phage, dose, route, bacterial target, barrier integrity, host condition and duration of exposure. Repeated high-dose exposure, inflamed mucosa and vulnerable populations may therefore justify more intensive immune and tolerability monitoring than short-term exposure in generally healthy adults.

Testable implication—If a host-support claim depends on immune modulation, predefined barrier or immune endpoints should be measured alongside phage recovery, target engagement and adverse event monitoring.

Finally, phage effects are conditional on formulation and delivery. A phage with suitable host range may still be ineffective if it loses viability during storage, is inactivated in gastric conditions, fails to reach the intended intestinal compartment or is incompatible with the product matrix [50,51,58,59]. Conversely, protective delivery systems may change site of release, local concentration and duration of exposure. In this sense, formulation is not merely a finishing step; it is part of the practical mechanism by which an oral phage product reaches the gut ecosystem.

The mechanistic argument for phagobiotics is therefore not that phages simply eliminate undesirable bacteria. It is that defined phages can, under appropriate ecological and technological conditions, exert selective pressure on bacterial populations and thereby reshape microbial community structure or function. This mechanism is promising precisely because it is specific, but it is also conditional. A credible phagobiotic claim should therefore connect phage identity, host susceptibility, formulation performance, microbiome context and functional endpoint at a depth appropriate to the intended use.

## 5. Human Evidence: Safety, Tolerability and Selectivity

Human evidence for gut-directed phage interventions remains limited. Available studies support feasibility and tolerability more consistently than efficacy and show that biological effects depend on target-host presence, susceptibility, formulation, dose and study design [22,27,28,29]. Neutral studies are therefore informative rather than merely unsuccessful: they distinguish gastrointestinal transit from target engagement and target engagement from a host-relevant benefit.

The PHAGE Study is one of the most relevant human studies for the phagobiotic concept. In this randomized, double-blind, placebo-controlled crossover trial, healthy adults with self-reported gastrointestinal distress consumed a commercial bacteriophage cocktail targeting *Escherichia coli*. The intervention was well tolerated and was associated with a reduction in fecal *E. coli* without broad disruption of overall microbiota diversity. The study also reported immune-related changes, including a decrease in circulating interleukin-4 [22]. For phagobiotics, the importance of this study lies less in any single clinical endpoint than in the demonstration that an oral phage cocktail can be evaluated in a non-therapeutic human setting using safety, tolerability and microbiome endpoints.

The PHAGE-2 study extended this line of investigation by examining supplemental bacteriophages in combination with the probiotic strain *Bifidobacterium animalis* subsp. *lactis* BL04. The design is relevant because future phagobiotic products may be combined with probiotics, prebiotics or other microbiome-directed ingredients. The study suggested that bacteriophages may modify the gastrointestinal ecological effects of probiotic supplementation, but it also illustrates the interpretive difficulty of combination products. When phages are administered with live microbial strains, observed outcomes cannot automatically be attributed to phage activity alone. Such designs require mechanistic endpoints capable of distinguishing direct phage effects, probiotic effects and phage–probiotic interactions [27].

The randomized pediatric diarrhea trial by Sarker and colleagues reported that oral coliphages were tolerated but did not demonstrate clinical benefit [28]. The study is instructive because insufficient baseline abundance of susceptible *Escherichia coli*, incomplete host coverage and mismatch between phage selection and patient microbiology can prevent target engagement even when dosing is feasible [28,60]. For product development, a neutral result therefore supports pre-screening for target presence, verification of susceptibility, and explicit distinction among formulation failure, absent target engagement and failure of target modulation to produce a functional benefit.

Earlier pediatric safety work with oral coliphages similarly supports the feasibility of oral administration while highlighting the need to interpret fecal phage recovery carefully [60]. Detection of phages in stool may indicate recovery after gastrointestinal transit, but it does not necessarily prove productive replication or meaningful ecological activity. Distinguishing passive transit from in situ amplification is therefore a methodological priority. Future studies intended to substantiate specific microbiome-modulation claims would benefit from quantitative phage recovery, target bacterial dynamics and, where relevant, resistance monitoring or phage–host linkage analysis [3,33,34].

Sterile fecal filtrate transplantation provides a complementary neutral example. In adults with metabolic syndrome, the intervention was tolerated and transiently altered phage composition and phage–microbe interactions, but it did not improve glucose metabolism [29]. Because the intervention was a complex virome-containing filtrate rather than a defined cocktail, it is not a phagobiotic product trial. It nevertheless shows that phageome modulation does not automatically yield a metabolic benefit and that microbiological and host-relevant endpoints must be evaluated separately.

Across human studies, safety and tolerability signals are more consistent than efficacy signals. This is not a weakness if interpreted correctly. For an emerging non-therapeutic microbiome category, early human evidence can establish that defined oral phage exposure is tolerated, that the product reaches the gut and that it does not broadly destabilize the microbiome under the conditions studied [22,28,29]. Broader functional or health-related endpoints should be interpreted only in relation to the claim, target population and supporting microbiological evidence.

Across neutral studies, three failure modes should be separated: insufficient delivery or infectivity, absence of a susceptible target, and failure of confirmed microbial modulation to produce a host-relevant benefit. This distinction guides whether the next iteration should change formulation, participant enrichment, phage composition or the proposed claim.

Future human studies should therefore be designed around the claim rather than around generic supplementation. A study designed to support a target-specific phagobiotic claim should prospectively define the target bacterial group, strain or function; verify its baseline presence when relevant; confirm susceptibility to the administered phage or cocktail when feasible; and specify microbiome endpoints that reflect the proposed mechanism [4,29,33,43]. These endpoints may include target abundance, strain-level replacement, resistance emergence, phage recovery, changes in microbial community structure, functional metagenomic changes, metabolite shifts or host-associated biomarkers. For gastrointestinal comfort or functional food positioning, symptom endpoints should be linked to microbiological and functional readouts rather than evaluated in isolation [15,16].

Study population selection is equally important. If the intended mechanism requires reducing a bacterial target, enrollment of participants lacking that target will dilute the apparent effect. If baseline microbiome state determines response, unstratified trials may obscure responder subgroups [3,33,34,35]. Enrichment designs, baseline microbiome stratification and pre-specified responder analyses may therefore be particularly useful for phagobiotic studies. These approaches should be used transparently and prospectively, not as post hoc explanations for weak results.

Current human studies support feasibility, tolerability and selective microbial effects more strongly than broad clinical efficacy or generalized microbiome-support claims. This evidence base is sufficient to justify further phagobiotic development, provided future products are evaluated through target-defined, microbiome-aware and claim-proportionate studies. Table 2 summarizes the human evidence base most relevant to the phagobiotic concept.

## 6. Preclinical and Translational Evidence for Phage-Mediated Gut Microbiota Modulation

Preclinical and translational models support mechanistic plausibility rather than direct human benefit. Animal, gnotobiotic, continuous fermentation, ex vivo colonic and formulation models are most useful when they connect a defined phage preparation to target engagement, community effects or delivery performance [4,43,61]. Detailed study characteristics are summarized in Table 3; the text below focuses on the cross-model lessons relevant to product design.

Targeting cytolysin-positive *Enterococcus faecalis* in alcohol-associated liver disease models illustrates function-level rather than species-level targeting. Phages reduced the defined bacterial target and attenuated disease phenotypes in humanized mice [61,62], supporting mechanistic precision but not a non-therapeutic human claim.

Subsequent work in related models has further suggested that oral phage cocktails can influence immune and tissue-associated outcomes, not only bacterial abundance. These findings should be interpreted cautiously because they arise from disease models rather than non-therapeutic microbiome-support settings. Nevertheless, they show that phage-mediated bacterial targeting can propagate to host-associated inflammatory and barrier-related pathways [4,41,43,57]. For phagobiotics, the broader lesson is that microbiological endpoints should be paired with functional readouts when a product claims to support host physiology.

Mouse intestinal colonization studies with carbapenem-resistant *Klebsiella pneumoniae* show that receptor choice, resistance evolution and strain-level susceptibility can determine decolonization outcomes [52,53,63]. Although these models are closer to therapy or infection control, they inform phagobiotic host range design and resistance monitoring.

Gnotobiotic and defined community models are especially useful because they allow phage effects to be observed in simplified but biologically interpretable ecosystems. In a model gut microbiome, phages have been shown to dynamically modulate bacterial community composition and metabolomic output [4,43]. Such systems make it possible to connect phage exposure to target bacterial changes, secondary microbial shifts and functional metabolites. Their limitation is also clear: simplified communities cannot capture the full variability and resilience of human gut ecosystems. Their strength is not prediction of population-level responses, but mechanistic tractability.

Defined microbial consortia and continuous fermentation systems provide complementary evidence. Studies using simplified human microbiota models, including SIHUMI-based systems, have shown that phage cocktails can reduce targeted bacteria and alter community behavior in controlled intestinal-like settings. These platforms are valuable because they allow repeated sampling, controlled environmental parameters and direct measurement of microbial dynamics over time [64]. They are particularly useful for testing whether a phage product affects only the intended target or produces secondary community shifts that may be relevant for safety or function.

An ex vivo colon model using an obesity-derived microbiota showed selective reduction in *Enterobacter cloacae* with preservation of overall community structure and changes in fermentation outputs [55]. This model is informative because target reduction, off-target community effects and functional readouts can be assessed together.

Fecal virome transfer studies show that complex viral fractions can influence bacterial recovery and taxa such as *Akkermansia muciniphila* in mice [65]. They are not phagobiotic product models, but they underscore both the ecological potential of the phageome and the analytical advantage of defined product composition.

Delivery studies show that manufacturing, storage, gastric acidity, bile and intestinal transit can reduce phage infectivity. Encapsulation, hydrogels and controlled-release systems may improve stability and site-specific recovery [50,51,58,59]. Such technologies are relevant when intestinal delivery is necessary for the proposed claim; they are not mandatory by default.

Across preclinical models, several principles emerge. The bacterial target should be defined at the appropriate resolution for the intended claim: species, strain, receptor type, resistance phenotype or functional trait. Host susceptibility should be confirmed when target-specific modulation is claimed [44,45,52,63,66]. Phage effects should be assessed in systems relevant to the expected use, including complex communities when community preservation or rebalancing is claimed [4,43]. Resistance and functional endpoints should be considered where they are biologically relevant. Formulation and delivery should be integrated into the experimental design when they determine whether phage–host contact occurs.

The most informative preclinical evidence for phagobiotics comes from models that connect a defined phage preparation with a plausible microbial target while remaining proportionate to the intended non-therapeutic claim. Preclinical success should not be measured only by bacterial killing but by the coherence of the evidence chain from phage identity to target modulation, community effect, functional output and translational plausibility. Table 3 summarizes the main model types that currently support this translational logic.

**Table 3:** Preclinical and translational models relevant to phage-mediated gut microbiota modulation.

Model or System	Phage-Related Intervention	Key Findings	What the Model Can Inform	Representative References
Alcohol-associated liver disease models	Phages targeting cytolysin-positive *Enterococcus faecalis*	Functional pathobiont reduction with attenuation of liver disease phenotypes and immune/tissue effects	Strong strain/function-level proof of concept; disease context limits non-therapeutic extrapolation	[41,57,61]
CRKP intestinal colonization models	Phages targeting carbapenem-resistant *Klebsiella pneumoniae*, including receptor-informed approaches	Intestinal target reduction/decolonization; resistance and receptor effects characterized	Supports target density, receptor diversity and resistance monitoring; closer to infection control territory	[52,53,63,67]
Defined or model gut microbiomes	Phage administration into simplified microbial ecosystems or SIHUMI-like consortia	Target reduction, community shifts and metabolomic effects under controlled conditions	Enables mechanism testing; simplified systems do not capture full human variability	[4,43,64]
Ex vivo human-derived colonic simulation	Two-phage cocktail targeting *Enterobacter cloacae* in obesity-derived microbiota	Selective target reduction with preserved community structure and altered SCFA profiles	Links target reduction, community preservation and function; lacks host immunity and long-term adaptation	[43,55]
Fecal virome transfer models	Virus-enriched fecal fractions rather than defined cocktails	Viral fractions influenced bacterial recovery or specific taxa such as *Akkermansia muciniphila*	Supports ecological capacity of gut viral communities; composition and mechanism are harder to control	[29,65]
Oral delivery and formulation models	Encapsulation, hydrogels, buffering or controlled-release systems	Improved phage protection, intestinal release or biological activity	Shows formulation can determine whether phage–host contact occurs; human confirmation is needed	[50,51,58,59]
Host range and receptor mapping assays	In vitro screening across target strain panels and receptor variants	Defines susceptibility, coverage, receptor diversity and resistance routes	Foundational for rational cocktails; in vitro host range may not predict in vivo access	[44,45,52,66]
Resistance monitoring models	Serial exposure and escape mutant characterization	Identifies resistance emergence and possible fitness, virulence or antibiotic susceptibility trade-offs	Helps interpret evolutionary steering; outcomes are context-dependent in complex communities	[52,53,63]

## 7. Safety and Manufacturing Quality Considerations for Phagobiotic Products

The safety assessment of phagobiotic products should be proportionate to their intended use. Phagobiotics, as defined in this review, are not proposed as medicinal products for the treatment or prevention of bacterial infection. They are also not intended to replace antibiotics or to eradicate pathogens in patients with disease. Their intended role is narrower: selective, non-therapeutic modulation of gut microbial communities using defined bacteriophages. This distinction is important because it affects the level and type of evidence that is scientifically appropriate.

Natural and controlled food-related exposure provides context but not a quantitative specification for a manufactured product [1,17,18,19,21,23,24,25]. Intentional supplementation may differ in dose, formulation, frequency, duration and population vulnerability. Safety assessment should therefore focus on the defined product, production host, impurities, stability, intended exposure and claim rather than assuming either class-wide safety or class-wide novelty.

For this reason, phagobiotic development should avoid importing the full logic of phage therapy medicinal products unless therapeutic claims are made. Medicinal phage products are commonly developed for infected or vulnerable patients, often under conditions where the target pathogen, route of administration, risk–benefit balance and clinical endpoints differ substantially from those of a food, supplement or functional microbiome product [5,6,7,32]. Phagobiotics require quality control, but the purpose of that control is different. It is to ensure that the consumer is exposed to the intended phages and not to unintended bacterial contaminants, residual production-host material, toxins, unstable formulations or poorly characterized mixtures.

Each phage component should be identifiable and traceable, and the final product should have controlled composition. Whole-genome sequencing supports identity, relatedness and screening for lysogeny functions, known toxin or virulence genes, antimicrobial resistance cargo and other undesirable mobile elements [26,30,31,32]. Genome analysis cannot prove safety because many phage genes remain unannotated; it reduces avoidable uncertainty. Where resident phages or prophages are central to the proposed mechanism, virome-aware baseline and follow-up analyses can help distinguish administered phages from background phageome dynamics.

Production-host control is likely to be one of the central quality issues for phagobiotics. Phages are propagated in bacterial cultures, and the production organism can contribute residual DNA, proteins, endotoxins, exotoxins, media components or other impurities if the manufacturing process is insufficiently controlled. Whenever feasible, production strains should be well characterized and suitable for reproducible manufacturing. If opportunistic or clinically relevant bacteria are used for propagation, the purification process should be designed to remove host-derived impurities to levels appropriate for oral use. In this respect, the critical safety concern is often not the phage particle itself, but the biological material that may accompany it after production [7,26,32,68].

Impurity control should be a core quality domain. For phages propagated on Gram-negative hosts, endotoxin measurement is particularly important, alongside residual host cell DNA and proteins, viable production bacteria, adventitious microorganisms, media components and process-related impurities [26,32]. No universally validated endotoxin limit exists for all oral non-therapeutic phage products, and parenteral K/M limits should not be transferred directly to oral use. A specification should therefore be justified for the route, maximum daily dose, duration, formulation, target population, jurisdiction and validated assay, with conservative control for repeated use and vulnerable populations.

Potency and stability requirements should also be proportionate. A phagobiotic product should contain viable phages at the level claimed on the label through the end of shelf life. For a simple product or a narrowly defined cocktail, total PFU may be adequate for some quality control purposes if the composition is otherwise controlled [26,32,69,70]. For more complex cocktails, component-specific monitoring may be needed to prevent one phage from dominating or disappearing during production or storage. The purpose is not to impose pharmaceutical complexity by default but to ensure that the marketed product remains comparable to the product used to generate safety, stability or microbiome data.

Formulation is another practical safety and quality issue. Phages may lose activity during drying, concentration, encapsulation, storage, exposure to excipients or passage through acidic gastric conditions [50,51,58,59]. A phagobiotic product does not necessarily need advanced targeted delivery unless the claimed effect depends on release at a specific intestinal site. However, it should demonstrate sufficient stability under the intended storage and use conditions. If gastrointestinal retention of infectivity is central to the proposed mechanism, simulated gastric and intestinal stability data become more important.

Host range characterization should follow the intended claim. Narrow-host-range phages may provide greater selectivity but require confirmation that relevant target strains are covered; broader-host-range phages or cocktails may improve population coverage but increase the need to assess off-target ecological effects and component-specific potency [35,44,45,46]. Single-phage products may be sufficient for a well-defined strain target, whereas cocktails may be justified by target heterogeneity or resistance risk. The required depth of strain-level testing should increase with the specificity of the modulation claim.

Horizontal gene transfer and transduction risk should be handled in the same proportionate way. These risks are relevant to phage biology, but they should not be presented as if every oral phage product carries a high or medicine-like risk. For well-characterized lytic phages used in non-therapeutic oral products, genomic screening, careful production-host selection and avoidance of obvious high-risk candidates may be sufficient for many product contexts. Additional empirical transduction studies may be justified when the production host carries undesirable genes, when the phage biology raises specific concerns, or when the intended use involves repeated high-dose exposure in vulnerable populations [26,30,31,67].

Microbiome endpoints are important for substantiating claims, but they should not be confused with basic safety requirements. If a product claims only identity, purity, oral intake and general exposure, extensive human microbiome studies may not be necessary. If the product claims selective modulation of a bacterial group, preservation of overall microbiota structure or support of a gut-associated function, then microbiome and functional endpoints become part of claim substantiation [4,22,43]. In other words, the evidence burden should increase with the specificity and biological ambition of the claim, not merely with the presence of phages in the product.

The same logic applies to immune monitoring and repeated exposure. Phages can interact with mucosal and systemic immunity, and anti-phage responses may be relevant in therapeutic contexts or repeated high-dose interventions [41,56,57]. For non-therapeutic phagobiotic use in generally healthy populations, routine medicinal-style immunogenicity packages may not be necessary unless there are specific risk factors, unusual formulations, vulnerable populations or long-term high-dose use. Tolerability data, impurity control and conservative claim language may be more relevant for early product development.

A phagobiotic product cannot be defined by total PFU alone. Each phage component requires identity, potency, genomic safety, impurity control and stability specifications, and these quality attributes must be connected to the intended microbiome effect. This is where phagobiotic development differs from loose natural product logic: the product is not merely a collection of phages but a controlled biological system designed to produce a defined ecological outcome. Table 4 summarizes the safety and quality control domains that should be considered for phagobiotic products.

## 8. Regulatory and Claim Boundaries

The translational status of a phage product is determined by intended use rather than by the phage particle alone. This principle is essential for phagobiotics. A bacteriophage may be used as a medicinal product, a food-processing antimicrobial, a veterinary intervention, an environmental biocontrol agent or a microbiome-directed ingredient. These uses may involve similar biological entities, but they do not share the same regulatory logic [6,7]. The relevant questions differ: whether the product treats disease, reduces contamination in a food matrix, supports a physiological function [71], or modifies a microbial ecosystem in a non-therapeutic context.

Food-use precedents should be interpreted precisely. In the United States, defined phage preparations have been evaluated as food additives or through generally recognized as safe (GRAS) notices for specified uses against bacterial pathogens in food [10,23,24]. These precedents demonstrate that controlled exposure to particular phage preparations can be acceptable under stated conditions; they do not establish class-wide GRAS status or substantiate gut microbiome-modulation claims.

The *Listeria*-specific phage preparation described in 21 CFR § 172.785 is particularly instructive. The regulation defines a purified bacteriophage preparation directed against *Listeria monocytogenes* and specifies quality-related attributes such as lytic-type phages, absence of lysogenic activity, potency requirements and sequence-based exclusions for certain undesirable bacterial elements [23]. This precedent is relevant not because it creates a phagobiotic pathway but because it shows that phage-specific identity and quality attributes can be formalized for a controlled food-related use. For phagobiotics, a comparable product-specific logic would be required, but the relevant endpoints would shift from reducing a pathogen in a food matrix to evidence appropriate to the intended non-therapeutic gut-directed claim [24,26,72,73].

GRAS notices for phage preparations should also be read as product- and use-specific. They may support the broader proposition that certain defined phage preparations can be considered safe for specified food uses under specified conditions [24,72,73]. They should not be cited as evidence that bacteriophages as a class are GRAS for repeated gut-directed microbiome modulation. The distinction is not semantic. A phage preparation applied to food to reduce *Salmonella*, *Shigella*, Shiga toxin-producing *E. coli* or *Listeria* before consumption differs from a phage cocktail intended to retain infectivity during gastrointestinal passage, interact with intestinal bacteria and support a microbiome-related function [26].

Medicinal product guidance is relevant but not directly transferable. Draft and emerging quality expectations for phage therapy medicinal products provide useful benchmarks for identity, production control, impurity management, potency and batch consistency [6,7,32]. These standards are valuable because they address phage-specific issues that conventional food or supplement frameworks may not fully capture. However, phagobiotics are not necessarily therapeutic products if they are not intended to treat, prevent or cure disease. The appropriate approach is therefore not to import the entire medicinal framework unchanged but to adapt selected quality concepts to a non-therapeutic microbiome context.

Illustrative jurisdictional overview—No dedicated phagobiotic pathway is assumed. In the United States, possible food, food additive/GRAS or dietary supplement routes depend on ingredient status and intended use; structure/function claims must be substantiated and cannot imply disease treatment. In the European Union, a novel food assessment may be relevant where there is no qualifying history of consumption, while health claims require separate scientific substantiation and authorization. For a product such as Listex™ P100, intended to prevent the presence of *Listeria monocytogenes* in ready-to-eat foods of animal origin, the Court of Justice of the European Union held that prior European Commission approval is required under Article 3(2) of Regulation (EC) No 853/2004 [74]. In the United Kingdom, novel food and food supplement rules may apply, and food claims cannot state or imply treatment, prevention or cure of disease. In each jurisdiction, medicinal classification remains possible when intended use or claims are therapeutic. This overview is descriptive rather than legal advice and cannot classify a specific product.

Claim boundaries are central and remain jurisdiction-specific. Illustrative microbiological wording such as “contains characterized phages active against selected *Escherichia coli* strains” describes product identity or in vitro activity but does not itself establish an in vivo benefit. A statement such as “selectively modulates a defined bacterial group” requires target-linked human microbiome evidence. Host-oriented wording such as “supports gastrointestinal comfort” requires appropriate human functional endpoints and may still require jurisdiction-specific authorization. Claims to treat infection, eradicate a pathogen, correct a diagnosed disorder or replace antibiotics imply therapeutic use and fall outside the proposed non-therapeutic framework [15,16]. These examples are an evidence ladder, not a list of legally pre-authorized claims.

The distinction between microbiological effect and health-related claim should be explicit. Demonstrating that a phage reduces a target bacterium in vitro does not substantiate a consumer claim. Demonstrating fecal recovery of administered phages does not substantiate microbiome modulation. Demonstrating reduction in a bacterial target in humans may support a selective modulation claim, but not necessarily a health benefit claim. Demonstrating improvement in gastrointestinal symptoms without confirming the microbial mechanism may support a symptomatic claim only if the regulatory context allows it, but it does not validate the proposed phagobiotic mechanism. The evidentiary package must match the claim [4,22,28,43]. For a selective microbiota-modulation claim, microbiological endpoints may be central. For gastrointestinal-comfort, barrier-support or other host-oriented claims, host-relevant endpoints become primary, while microbiological data provide mechanistic support [15,16].

This proportionality is especially important for functional foods and dietary supplements. These categories often operate near the boundary between physiological support and disease treatment. Phagobiotics would add another layer of complexity because the active entities are viruses whose effects depend on bacterial hosts. Conservative wording is therefore not merely regulatory caution; it is scientifically accurate. Claims should be anchored to the demonstrated effect of the defined product in the intended population [15,16,71]. If the evidence shows safety, identity, purity and transit, the claim should not imply selective modulation. If the evidence shows target reduction without functional benefit, the claim should not imply improved health. If the evidence shows target reduction plus a validated functional endpoint, the claim can be broader but should remain non-therapeutic unless the product is developed as a medicine.

## 9. Proposed Evidence Framework for Phagobiotic Development

The development of phagobiotics requires an evidence framework that integrates phage biology, microbiome ecology, product quality and claim substantiation. A phage therapy framework may be overly focused on disease treatment for products without therapeutic claims [5,6], whereas a food biocontrol framework focuses on pathogen reduction in food matrices [8,9]. Consumer health frameworks may not fully capture host range dependence, cocktail composition, phage stability or phageome-level ecological effects [26]. A layered model should therefore link product design to biological activity, host-relevant outcomes and claim language [15,71].

**Figure 3:**
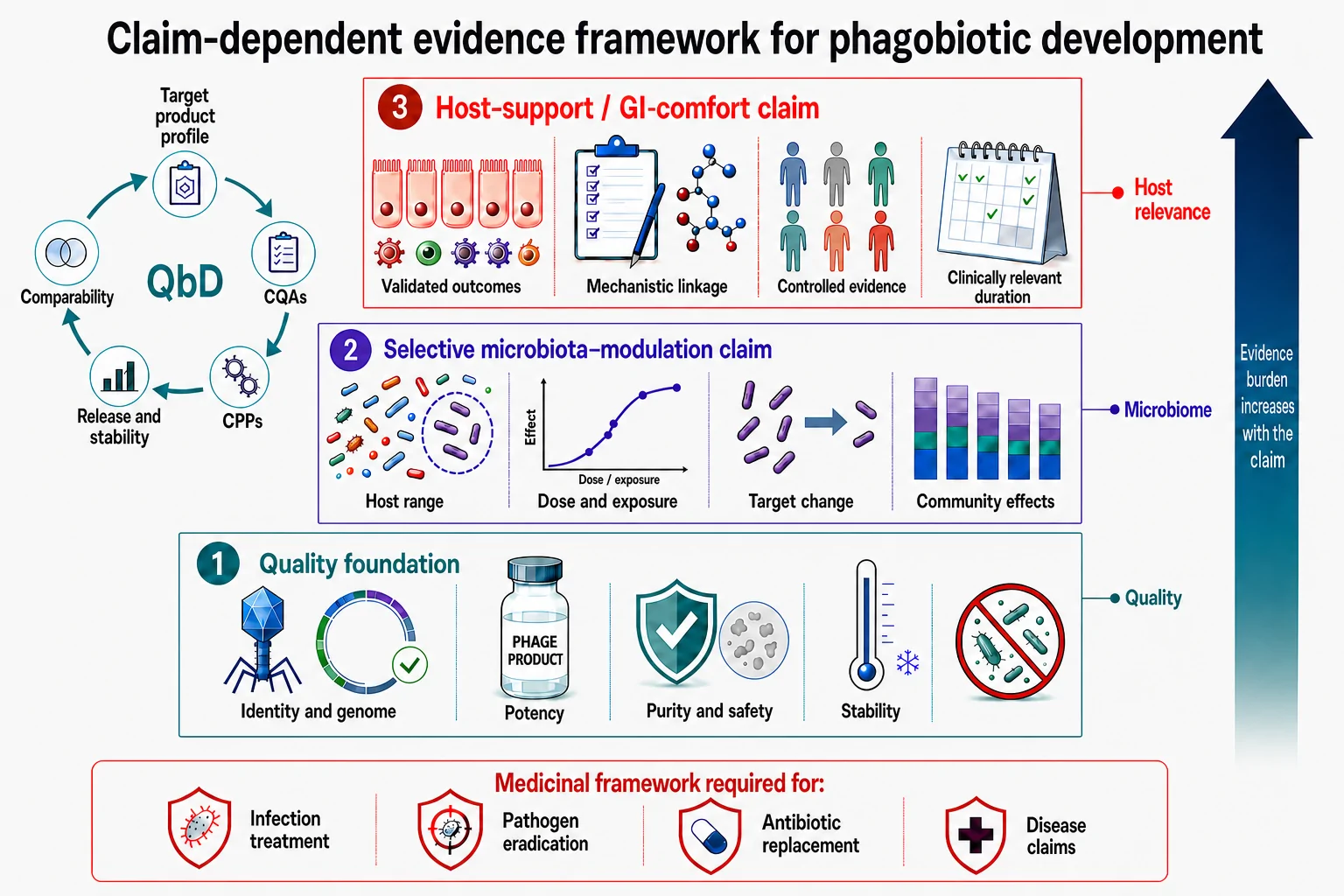
Claim-dependent evidence framework for phagobiotic development. Defined phagobiotic products require a quality foundation for all uses, including identity, defined composition, production-host control, impurity removal, absence of viable production bacteria, stability and label accuracy. When selective microbiome modulation is claimed, additional characterization becomes relevant, including host range rationale, target bacterial group, gastrointestinal stability and infectivity, where relevant, and microbiome endpoints. When host-support or gastrointestinal-comfort claims are made, the evidence burden increases further and may require tolerability data, symptom-related or functional endpoints, and claim-linked human or real-world evidence. Boundary conditions remain non-therapeutic: no infection-treatment, pathogen-eradication or antibiotic-replacement claims unless the product is developed under an appropriate medicinal framework.

A quality-by-design approach provides a useful organizing principle for this framework, but it should be applied as a claim-dependent tool rather than as a default medicinal development template (Figure 3) [75,76,77]. Development should begin with a clear intended use, target population, product format, dose range and claim boundary. These intended product characteristics should determine which quality attributes are critical for a given use. For most non-therapeutic phagobiotic products, the common foundation would include product identity, manufacturing purity, production-host control, stability and label accuracy. Additional attributes such as detailed host range mapping, component-specific potency, transduction testing or advanced microbiome endpoints become more important as the claim becomes more specific or biologically ambitious.

The first evidence layer is product definition and manufacturing quality. A defined phagobiotic product should be traceable and compositionally controlled, with a manufacturing process designed to limit production-host residues, viable production bacteria and process-related impurities. Genome sequencing can support identity and exclude obvious undesirable features, but the key point for non-therapeutic use is not medicinal-style proof of safety; it is reductions in avoidable uncertainty and preservation of product transparency [26,30,31,32].

The second evidence layer is formulation and comparability. Manufacturing controls should include production host selection, propagation conditions, purification, concentration, formulation, storage and packaging [7,26,32,50,51]. Product quality should include purity, label-relevant viability or potency, stability under intended storage and use conditions, and matrix compatibility. Comparability remains important: evidence generated with one composition, production host, purification method, formulation or dose should not be assumed to support a materially different product unless relevant quality attributes and intended use remain comparable.

The third evidence layer is microbiological and functional substantiation. Before claims of selective microbiome modulation are made, the candidate product should be evaluated in systems that match the intended mechanism of action. These may include in vitro host range assays, mucosal models, continuous fermentation systems, ex vivo colonic simulations, defined microbial consortia, gnotobiotic models or humanized animal models [43,55,59]. The choice of model should be driven by the intended biological effect. A product intended to modulate a bacterial group should demonstrate a plausible connection between phage composition and target modulation. A product intended to modulate a metabolic microbial function should include functional readouts such as metabolite profiles. These data establish biological plausibility and mechanisms, but they do not by themselves substantiate host–benefit claims.

The fourth evidence layer is host-relevant substantiation. This layer becomes particularly important when phagobiotic claims are framed not as “modulates bacterium X” but as support for a physiological or experiential outcome, such as gastrointestinal comfort, tolerability or maintenance of a normal gut-associated function. In such cases, microbiological endpoints are necessary to support the proposed mechanism, but host-relevant endpoints become decisive for claim substantiation. These may include gastrointestinal symptom scores, stool consistency, tolerability measures, predefined inflammatory markers, barrier-associated biomarkers or other claim-linked readouts [4,22,27,28,29]. The endpoint hierarchy should be explicit: microbiological outcomes are primary for microbiome-modulation claims, whereas host-relevant outcomes are primary for host-support or comfort claims.

The fifth evidence layer is human evidence, interpreted proportionately. Early human studies may prioritize safety, tolerability, target-host dependence and microbiome selectivity. A study designed to support a target-specific claim should prospectively define the bacterial target or microbial function, verify baseline presence of susceptible hosts where relevant, and distinguish passive phage transit from productive ecological activity. Microbiological endpoints may include quantitative changes in target bacterial groups, preservation of overall community structure, emergence of phage resistance, phage recovery and virome-aware phage–host interaction analysis [22,27,28,29,33]. However, the scope of human evidence should be proportional to product novelty, intended population, exposure pattern and claim breadth. Controlled human studies may be necessary for a new product platform, new population, new host-oriented claim or materially changed phage cocktail. They may not be scientifically necessary for every closely related product iteration if comparability to an already substantiated platform is demonstrated and the intended claims remain proportionate.

The sixth layer is claim substantiation. Claims should be proportional to the evidence generated and to the degree of uncertainty remaining. If a product has demonstrated only identity, purity, tolerability and gastrointestinal transit, claims should not imply selective microbiome modulation. If it has demonstrated selective reduction in a predefined bacterial group without community disruption, claims may refer to selective microbiota modulation, provided the population and target are specified. If host-relevant endpoints are demonstrated in controlled human studies or justified through a robust comparability package, broader gastrointestinal comfort or microbiome-support claims may be considered, but therapeutic claims should remain excluded unless the product is developed under a medicinal framework [15,16].

This framework is therefore better understood as a claim-dependent evidence model than as a rigid linear clinical development pathway. Human findings may require reformulation, cocktail redesign, host range expansion or refinement of target populations. Manufacturing constraints may reshape which phages are suitable for development. Claim boundaries may determine whether microbiological endpoints are sufficient or whether host-relevant endpoints are required. A quality-by-design model is useful because it treats product definition, manufacturing controls, biological mechanism, comparability and evidence generation as interconnected. Under this model, phagobiotics would be developed not as generic phage mixtures but as defined microbiome-directed products with proportionate evidence requirements.

Worked QbD example (hypothetical)—Consider an oral two-phage product intended to selectively reduce a predefined *Escherichia coli* subgroup in target-positive healthy adults without therapeutic claims. The target product profile would specify population, oral dose, duration, target subgroup and microbiological claim. Critical quality attributes would include component identity, strictly lytic genomes without known undesirable cargo, component-specific potency, purity, absence of viable production bacteria, justified endotoxin specification, formulation stability and label accuracy. Critical process parameters would include production-host state, fermentation conditions, harvest timing, purification, blending and encapsulation. Release and stability data would be linked to gastrointestinal infectivity, target engagement and preservation of overall community structure. A material change in host strain, purification, component ratio or formulation would trigger a comparability assessment before prior evidence was bridged. The claim-dependent evidence requirements are summarized in Table 5.

## 10. Conclusions and Future Directions

Bacteriophages are integral components of the gut microbiome and phageome, not merely external antibacterial agents. Their host-dependent and ecologically embedded interactions provide a plausible basis for targeted microbiota modulation. The phagobiotic framing proposed here distinguishes a defined non-therapeutic, microbiome-directed product from phage therapy and food biocontrol while recognizing that the term is not an established regulatory category.

The strongest argument for the phagobiotic concept is not any single clinical trial but the convergence of several evidence layers. Humans are naturally exposed to diverse phages through the gut ecosystem, early-life colonization, breast milk and food matrices [1,17,18,19]. Experimental models show that phages can reduce defined pathobionts or antimicrobial-resistant colonizers, reshape microbial community structure, influence metabolic outputs and affect host-associated inflammatory or barrier-related readouts in model systems [43,55,61,63]. Human studies remain limited, but they support the feasibility and tolerability of oral or gut-directed phage exposure and provide evidence for selective microbial effects under defined conditions [22,27]. Together, these data support biological plausibility, but they do not yet establish phagobiotics as a mature category with broadly proven health benefits.

A key future direction is the transition from generic phage administration to target-defined microbiome modulation [43,44,45,46,61]. Phagobiotic development should not begin with the assumption that adding phages to the gut is beneficial. It should begin with a clearly defined microbial target, ecological state or functional endpoint. The target may be a pathobiont, an antimicrobial-resistant colonizer, a strain carrying a harmful functional trait, or a microbial activity associated with gastrointestinal, inflammatory or metabolic phenotypes. In each case, the intended effect should be measurable, the susceptible bacterial host should be detectable when relevant, and the phage product should be designed around a target rationale appropriate to the intended claim.

Human trials will be decisive for claims that go beyond product identity, tolerability or general oral exposure. Future studies intended to support selective microbiome-modulation claims should define target bacterial groups, verify baseline presence of susceptible hosts where relevant, quantify target reduction, assess preservation of overall microbiota structure, monitor phage recovery when informative, and include functional readouts such as short-chain fatty acids, inflammatory markers, barrier-associated biomarkers or gastrointestinal symptom scores when these endpoints are linked to the intended claim [4,28,29,33,43]. Stratified or enrichment designs may be particularly important because phage activity is conditional on host availability and susceptibility.

Safety and manufacturing quality should remain central, but the framework should be proportionate to non-therapeutic use. Natural phage exposure and controlled food-use precedents support a lower level of intrinsic toxicological concern for oral exposure to well-characterized phages than would apply to many entirely novel biological entities. The main product-specific risks are more practical: undefined composition, unsuitable or poorly controlled production hosts, residual bacterial material, endotoxins or other process-related impurities, loss of activity during storage, and claims that exceed the evidence [26,30,31,32,75]. For this reason, the core quality foundation for phagobiotics should focus on product identity, purity, production-host control, impurity removal, stability under intended conditions of use and label accuracy. Additional testing, including detailed host range analysis, transduction assessment, microbiome endpoints or controlled human studies, should be driven by the intended claim, target population, dose, duration of use and product novelty.

Several scientific gaps remain. Standardized methods are needed for phage–host linkage, host range testing, resistance monitoring and virome-aware endpoint assessment [3,33,34,45,66]. More work is needed to understand how phage interventions interact with diet, probiotics, antibiotics, mucosal immunity and baseline microbiome state [35]. Formulation science must be integrated with microbiome biology because phage infectivity and localization in the gut determine whether the intended ecological interaction can occur [50,51]. Future human studies should clarify which populations are most likely to benefit from phagobiotic strategies, which microbiological endpoints are sufficiently reproducible, and which host-relevant endpoints are robust enough for non-therapeutic claims. As product platforms mature, comparability and bridging principles should be defined so that evidence generation remains rigorous without defaulting to full de novo clinical evaluation for every controlled product iteration.

Prioritized research questions—Early development should determine the minimum dose that preserves infectivity at the intended intestinal site; identify which baseline target, susceptibility and phageome features predict response; validate phage–host linkage and resistance-monitoring methods; and separate passive transit from productive target engagement. Claim-linked biomarkers of barrier function, immune response and microbial metabolism also require validation.

Tractable early populations—Initial non-therapeutic studies are most interpretable in adults with a predefined, detectable and susceptible bacterial target, stable background therapy and measurable microbiological endpoints. Pediatric, immunocompromised or actively inflamed populations may be scientifically important but require stronger safety justification and should not be treated as the default first-use population.

The phagobiotic concept should therefore be advanced with both ambition and restraint. Its ambition lies in the possibility of developing a new class of precision microbiome modulators based on the natural ecological specificity of bacteriophages. Its restraint lies in recognizing that natural exposure, food-use precedents and preclinical success do not substitute for product quality or claim-linked evidence. The field will progress most credibly if phagobiotics are evaluated as defined non-therapeutic products with clear microbial targets, controlled manufacturing, proportionate endpoint hierarchies, conservative claim framing and transparent safety monitoring.

Phagobiotics are best understood as an emerging platform, not an established category: their promise depends on precise product design, controlled manufacturing and careful evidence generation. If developed through a framework that integrates phage biology, quality-by-design principles, microbiome ecology, host-relevant endpoints, comparability logic and proportionate claims, phagobiotics may become a distinct and credible category within microbiome-directed interventions.

## Figures and Tables

**Table 1:** Natural routes of human exposure to bacteriophages.

Route or Source	Main Phage-Related Features	Relevance to Phagobiotics	Interpretation Caveats	Representative References
Resident gut virome	Phages are major components of the gut viral community, including virions, prophages and active populations	Establishes phages as normal ecological participants in the gut microbiome	Natural presence does not demonstrate product-specific safety or efficacy	[1,2,3]
Early-life colonization and persistence	Phages emerge during microbiome assembly and some infant gut phage strains persist over time	Shows that phage–bacterium interactions can be durable and developmentally structured	Does not justify pediatric supplementation without dedicated evidence	[17,20]
Human milk	Human milk contains microbial and viral components, including bacteriophages	Links phage exposure to a physiological nutritional interface	Evidence is heterogeneous and method-dependent	[18]
Foods and food metagenomes	Phages accompany food-associated bacterial communities and are underdetected by culture alone	Supports repeated dietary exposure to diverse phage populations	Detection does not establish viability or post-ingestion biological effect	[19,21]
Fermented and dairy foods	Phages interact with starter and ripening microbiota and may shape fermentation dynamics	Demonstrates structured food-associated phage ecology	Industrial fermentation ecology is not intentional gut modulation	[36,37,38,39,40]
Food biocontrol phages	Defined phage preparations have been evaluated or accepted for specific pathogen control uses in foods	Provides controlled food exposure precedents that help contextualize safety of defined oral phage exposure while remaining use-specific	Food biocontrol is product- and use-specific and is not equivalent to gut-directed phagobiotic use	[10,23,24,25]

**Table 2:** Human gut-directed phage intervention studies relevant to the phagobiotic concept.

Study/Intervention	Population, Design and Intervention	Main Findings	Relevance to Phagobiotics	Main Caveat	Representative References
PHAGE Study	Randomized, double-blind, placebo-controlled crossover trial in adults with self-reported gastrointestinal distress; oral *E. coli*-targeting cocktail	Fecal *E. coli* reduction; no broad microbiota disruption reported; generally well tolerated	Strongest directly relevant non-therapeutic human phage study	Exploratory endpoints; does not establish broad gut health efficacy	[22]
PHAGE-2	Human study of *Bifidobacterium animalis* subsp. *lactis* BL04 with or without supplemental phages	Suggested modification of probiotic-associated gut ecological effects; tolerability supported	Relevant to combination microbiome products	Effects cannot be attributed to phages alone	[27]
Pediatric oral coliphage trial	Children with acute bacterial diarrhea; oral coliphage preparation	Gastrointestinal phage exposure observed; safe, but no clinical benefit	Shows that safe transit does not equal efficacy	Disease setting; target-host coverage was insufficient	[28]
Earlier pediatric coliphage safety work	Pediatric oral coliphage administration	Dose-related fecal recovery and tolerability	Supports interpretation of oral transit and recovery	Fecal recovery does not prove in situ amplification or ecological activity	[60]
Sterile fecal filtrate transplantation	Adults with metabolic syndrome; viral/phage-containing sterile fecal filtrate	Transient viral and phage–microbe changes; well tolerated; no glucose metabolism benefit	Supports human manipulability of gut viral communities	Not a defined phagobiotic product	[29,33]

**Table 4:** Proportionate safety and manufacturing quality considerations for phagobiotic products.

Domain	Core Consideration	Why It Matters	When Additional Evidence May Be Needed	Representative References
Product identity	Defined phage preparation; traceable composition	Confirms that the consumer receives the intended product rather than an undefined natural mixture	Complex cocktails, platform bridging or changes in composition	[26,30,32]
Production host	Characterized propagation strain and controlled process	The production host is a major source of residual biological impurities	Opportunistic or clinically relevant production hosts; major process changes	[7,26,32,68]
Purity	Control of viable production bacteria, endotoxin, host DNA/protein, media residues and process impurities	Principal product-specific safety concern for manufactured oral phage preparations	High-dose or repeated use; vulnerable populations; Gram-negative production hosts	[26,32]
Stability	Maintenance of claimed viable phage level through shelf life	Supports label accuracy and batch consistency	Sensitive formulations, complex matrices or long storage conditions	[26,32,50,51]
Genomic characterization	Identity confirmation and screening for obvious undesirable genes	Reduces avoidable uncertainty and supports transparency	Novel phages, complex cocktails or specific target claims	[26,30,31,32]
Host range	Characterization proportional to intended claim	Connects product composition to the proposed microbial effect	Claims about selective reduction in or modulation of a defined bacterial group	[35,44,45,46]
GI stability and infectivity	Stability under relevant gastrointestinal conditions when gut activity is claimed	Connects formulation to intended site of action	Site-specific intestinal release claims or products with acid-sensitive phages	[50,51,58,59]
Microbiome endpoints	Claim-linked microbiome assessment	Supports claims of modulation, not basic product identity or purity	Selective modulation or microbiome-support claims	[4,22,43]
Human/tolerability data	Evidence proportional to exposure pattern and claims	Supports consumer use and substantiation of host-relevant claims	New product class, repeated use, host-support claims or vulnerable populations	[22,27,28,29]
Claim boundaries	Non-therapeutic positioning	Prevents drift into medicinal claims	Infection-treatment, pathogen-eradication or antibiotic-replacement claims	[15,16,71]

**Table 5:** Claim-dependent evidence framework for phagobiotic development.

Claim/Use Level	Appropriate Evidence Focus	Examples of Supporting Data	What Should Not Be Inferred	Representative References
Defined oral phage product	Identity, purity, production-host control, stability and label accuracy	Defined composition; absence of viable production bacteria; impurity control; shelf life data	Selective microbiome modulation or health benefit	[26,32,68]
General oral phage exposure or non-specific positioning	Safety rationale from natural exposure and food-use precedents plus product quality	Gut virome, food and milk exposure literature; GRAS/food-use precedents; manufacturing controls	Specific microbiome or host benefit	[1,17,18,19,21,23,24,25]
Selective microbiome modulation	Target rationale, host range characterization, GI stability/infectivity and microbiome endpoints	Reduction in or modulation of a defined bacterial group; preservation of community structure	Clinical or symptomatic benefit	[22,43,55]
Functional microbiome support	Microbiome endpoints plus functional readouts	SCFAs, metabolomics, functional metagenomics, barrier-related or inflammatory markers as relevant	Disease treatment or prevention	[4,43,55]
GI comfort or host-support claim	Human tolerability and claim-linked host-relevant outcomes	Symptom scores, stool parameters, tolerability and relevant biomarkers	Treatment of IBS, infection, dysbiosis or inflammation	[15,16,22,27,28,29]
Therapeutic claim	Medicinal framework rather than phagobiotic positioning	Clinical endpoints, infection outcomes, pathogen eradication or antibiotic replacement evidence	Non-medicinal positioning	[5,6,7,32]

## Data Availability

No new data were created or analyzed in this study. Data sharing is not applicable to this article.

## Abbreviations

ARG, antimicrobial resistance gene; BAM, bacteriophage adherence to mucus; EFSA, European Food Safety Authority; EMA, European Medicines Agency; FDA, U.S. Food and Drug Administration; GI, gastrointestinal; GRAS, generally recognized as safe; HGT, horizontal gene transfer; QbD, quality by design; SCFA, short-chain fatty acid; WGS, whole-genome sequencing.
